# A civilian perspective on ballistic trauma and gunshot injuries

**DOI:** 10.1186/1757-7241-18-35

**Published:** 2010-06-17

**Authors:** Philipp Lichte, Reiner Oberbeck, Marcel Binnebösel, Rene Wildenauer, Hans-Christoph Pape, Philipp Kobbe

**Affiliations:** 1 Department of Trauma Surgery, University Hospital of the RWTH Aachen, Aachen, Germany; 2 Department of Trauma Surgery, University of Essen, Essen, Germany; 3 Department of Surgery, University Hospital of the RWTH Aachen, Aachen, Germany; 4 Department of Surgery, University of Würzburg, Würzburg, Germany

## Abstract

**Background:**

Gun violence is on the rise in some European countries, however most of the literature on gunshot injuries pertains to military weaponry and is difficult to apply to civilians, due to dissimilarities in wound contamination and wounding potential of firearms and ammunition. Gunshot injuries in civilians have more focal injury patterns and should be considered distinct entities.

**Methods:**

A search of the National Library of Medicine and the National Institutes of Health MEDLINE database was performed using PubMed.

**Results:**

Craniocerebral gunshot injuries are often lethal, especially after suicide attempts. The treatment of non space consuming haematomas and the indications for invasive pressure measurement are controversial. Civilian gunshot injuries to the torso mostly intend to kill; however for those patients who do not die at the scene and are hemodynamically stable, insertion of a chest tube is usually the only required procedure for the majority of penetrating chest injuries. In penetrating abdominal injuries there is a trend towards non-operative care, provided that the patient is hemodynamically stable. Spinal gunshots can also often be treated without operation. Gunshot injuries of the extremities are rarely life-threatening but can be associated with severe morbidity.

With the exception of craniocerebral, bowel, articular, or severe soft tissue injury, the use of antibiotics is controversial and may depend on the surgeon's preference.

**Conclusion:**

The treatment strategy for patients with gunshot injuries to the torso mostly depends on the hemodynamic status of the patient. Whereas hemodynamically unstable patients require immediate operative measures like thoracotomy or laparotomy, hemodynamically stable patients might be treated with minor surgical procedures (e.g. chest tube) or even conservatively.

## Introduction

In contrast to a stagnating incidence of civilian gunshot wounds in the United States, gunshot violence shows different trends in European countries. Firearm associated crime was increasing up to 30% in the UK between 1998 and 2002 [1]. In the same period firearm associated crime in Germany was markedly decreasing. In 2007 in Germany only 4558 criminal acts with the use of firearms were registered [2]. Additionally, in high income countries a significant number of gunshot wounds are related to suicide attempts [3]. As seen in the United States, gunshot violence has besides its medical importance also an enormous economic impact as the third most costly etiology of injury and the fourth most expensive form of hospitalization [4-6]. Therefore, treatment algorithms for emergency care of gunshot injuries have to be established in European trauma departments. An understanding of general ballistic principles is of major importance to guide clinical management of patients with gunshot injuries.

## Methods

This article bases on a literature search of the National Library of Medicine and the National Institutes of Health MEDLINE database using PubMed http://www.pubmed.gov. Search terms have been "gunshot injury", "penetrating injury" and "ballistic trauma". Articles about penetrating trauma which mainly deal about stab injuries were eliminated. Additionally the content is based on the personal experience of the authors, achieved by working in level one trauma centers in Germany and the United States.

### Ballistics

Firearm injuries are generally classified as low- or high-velocity injuries. Low-velocity wounds are attributed to projectiles with muzzle velocity of less than 600 meter per second (m/s), are classically caused by handguns and are therefore more common in the civilian population. The injury is usually less severe as compared with high-velocity wounds, which are caused by military or hunting weapons with a muzzle velocity of more than 600 meter per second.

**Figure 1:**
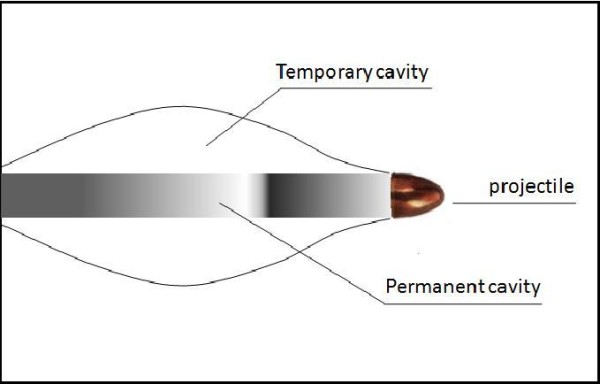
**The mechanism of cavitation can cause tissue destruction along the bullet diameter**.

Two areas of projectile-tissue interaction have to be differentiated: the permanent and the temporary cavity [7]. In low- velocity bullets the direct tissue destruction with its localized area proportional to the size of the projectile plays the major role, whereas in high-velocity injuries the lateral tissue expansion ("cavitation") becomes more important. After passage of the projectile there is a transient lateral displacement of tissue which can reach the 10 to 40-fold diameter of the bullet (Fig. 1). If the projectile crosses elastic tissue, such as skeletal muscle, blood vessels and skin, this tissue may be pushed aside after passage of the bullet, but then rebound. In cases of inelastic tissue, such as bone and liver, fractures and tissue destruction can be the consequence [7].

Although muzzle velocity is clearly an important aspect of the missile's wounding potential, tissue trauma is related to the degree of energy transfer [8], which depends on several factors [9]:

• Projectile velocity (low- vs. high-velocity)

• Entrance profile (in which degree the bullet enters the body)

• Caliber of projectile

• Design of the projectile

• Distance traveled within the body (penetrating projectiles deliver their total kinetic energy to the body, whereas perforating projectiles transfer significantly less)

• Biologic characteristics of the impacted tissue

• Mechanisms of tissue disruption (e.g. stretching, tearing, crushing)

Shotguns for example, which principally are considered as low-velocity weapons, are responsible for substantial injuries resulting in a mortality rate nearly twice that attributed to other firearms [10-12]. Further, the design of the bullet plays an important role [13]. Different types of bullets are described in table 1.

**Table 1:** Design and effects of different types of bullets [13].

Full Metal Jacket ammunition	A metal casing around a lead core	These bullets are dimensionally stable and produce non-expanding and deep penetrating wounds.
Jacket Hollow Point ammunition	Bullets with an exposed, hollowed lead tip which allows expansion on the impact.	Tissue penetration is less deeply than in Full Metal Jacket ammunition but more energy is transferred to the tissue.

Soft Point ammunition	An exposed lead tip causes a rapid expansion of the bullet on impact at lower velocities.	This rapid expansion is responsible for wounds which are significant wider than the diameter of the bullet.

Altered ammunition	Ammunition can be altered to increase the severity of injury. An infamous example is the Dum Dum projectile, produced by cutting a cross in the soft lead tip of the bullet.	This modification ensures that the bullet will fragment at the impact. Dum dum projectiles are responsible for very high energy transfer to the tissue and therefore tall inner wounds. They are banned for usage in war by an amendment of the Geneva Convention.

Hollow- and soft pointed ammunition are often used by huntsman and police forces.

These bullets leave the body with less kinetic energy or usually stop in the body. Therefore they are posing a smaller risk to bystanders.

### Initial assessment

After reaching the emergency department every patient should be treated according to the ATLS^® ^guidelines. The ABC structured physical examination provides a secure scheme to quickly identify immediately life threatening conditions [14]. For academic reasons the chapters are structured into anatomical regions.

### Head and neck

**Figure 2:**
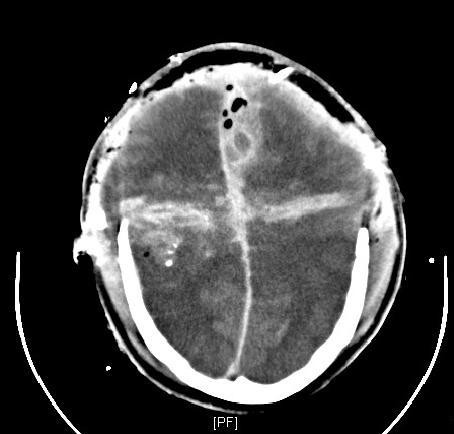
**CT-scan of a lethal gunshot injury of the brain with massive swelling and increased pressure despite of craniotomie**. The patient died despite of immediate craniotomie.

Penetrating craniocerebral injuries are associated with a high lethality especially after attempted suicide. The mortality rate is described up to 88% [15]. 80-90% of these patients die within the first 48 hours [16,17]. The Glasgow-Coma-Scale shows a high correlation to the extent of the injury [18,19]. After stabilizing the vital parameters of the patient a cranial CT scan can show the cerebral damage (Fig. 2). CT angiography can give additional information about vascular injuries.

An aggressive operative procedure with removal of all foreign bodies and debridement of the wound path seems to have no benefit for the outcome and therefore a general removal of the bullet is not recommended [20]; however dura leakages should be closed in order to avoid secondary infections [21]. More controversial is the treatment concept for gunshot related intracranial haematomas. Some authors had recommended an evacuation of haematomas [22,23], whereas others prefer evacuation only in cases of elevated intracranial pressure (ICP) or mass effects [24,25]. For the interventional measuring of the intracranial pressure there is also no evidence based recommendation. The advantage of an ICP monitoring in patients with altered level of consciousness is to have the possibility to decompress early in cases of an increasing ICP [21].

Gunshot injuries to the neck are feared due to the physical proximity of important anatomical structures. From the anatomical point of view the injuries are divided in 3 zones: Zone I from the clavicle to the cricoid, zone II from the cricoids to the jaw angle and zone III from the jaw angle to the skullbase. Because bleeding from the greater vessels of the neck can be quickly life threatening, the bleeding has to be stopped immediately. In some cases the ligation of the vessel can be necessary. Besides the laryngoscopy a CT angiography can uncover injuries of relevant organs (trachea, larynx, pharynx) and closed vascular injuries (pseudoaneurysms, thrombosis) [26]. Endovascular methods gain an increasing importance also in the treatment of injuries of the neck vessels, especially pseudoaneurysms [27]. An operative exploration is indispensable if relevant organs are injured. Otherwise a conservative treatment can be considered, especially for zone II injuries [28].

### Thorax injuries

The incidence of simultaneous injuries of thorax and abdomen ranges between 6%-42% [29,30]. Gunshot injuries to the chest are associated in 34% to 36% with haemato- or haematopneumothorax [31]. The high degree of energy in projectiles causes a high prevalence of lung contusion around the trajectory (43%) [31] and associated diaphragmatic injuries occur in 59% [29,32]. Cardiac injuries are rare in patients who reach the hospital because these injuries are often lethal at the scene [31,33].

**Figure 3:**
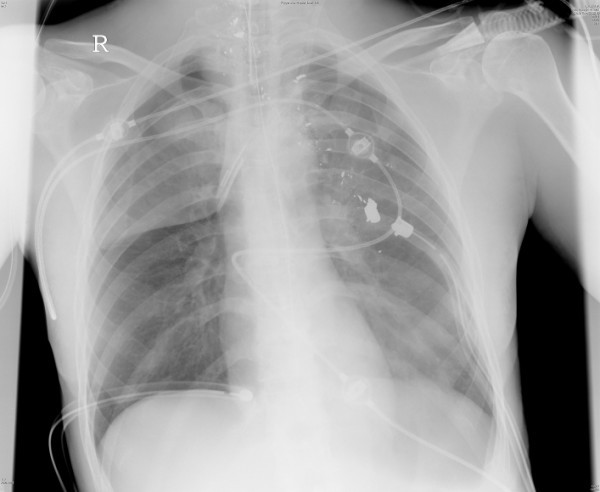
**Bilateral chest tubes have been inserted after gunshot injury**. The bilateral haematopneumothrax have been drained sufficient.

The most common life threatening injuries of the thorax are haemato- or haematopneumothorax, tension pneumothorax and pericardial tamponade. They should be diagnosed within the first physical examination and be treated immediately. If the patient is in a stable condition a chest X-ray is helpful to show the expansion of the lung and mediastinum [14]. The second standard investigation should be an ultrasound examination (Focused Assessment with Sonography for Trauma (FAST)) which can help to identify a pericardial tamponade [34]. The most important therapeutic intervention is the insertion of a chest tube [35-37], which is indicated in all cases of pneumothoraces larger than 2 cm and haematothoraces extending over the seventh rib [31] (Fig. 3).

CT diagnostics often allow the delineation of the precise injuries and can determine the need for therapeutic interventions or the need for further diagnostics. CT-scans can show the trajectory of mediastinal injuries in 75% [38] and if mediastinal injuries are assumed the CT scan should be completed with additionally angiography, esophagoscopy, barium swallow, and bronchoscopy [39]. Thoracoscopy has gained wider acceptance since it is more sensitive for small diaphragmatic lesions as compared to CT-scans and it easily allows diaphragmatic repair in hemodynamic stable patients [40,41].

The management algorithms for gunshot injuries to the chest are very similar in most trauma centers indicating that most gunshot injuries can be managed successfully without explorative thoracotomy [36,37,42]. Indications for thoracotomy however are: clinical or echocardiographic evidence of cardiac tamponade, unstable cardiac circulation, or a chest tube delivering more than 1 to 1.5 liters of blood immediately after the insertion or continued bleeding of more than 200 ml/h for 3 hours [31]. 85% of all pulmonary injuries which requires operation can be managed successfully with stapled pulmonary tractotomy [43].

Previously it has been recommended to perform an operative exploration of any patient with transmediastinal gunshot injuries. Due to the high rate of negative thoracotomies, nowadays more sophisticated diagnostics are used to identify those patients who benefit from thoracotomy [43].

### Abdominal injuries

In contrast to the treatment of gunshot injuries to the chest, treatment protocols of abdominal gunshot injuries underlie to a lower degree of generally accepted standards. Formerly, every patient with abdominal gunshot wounds underwent laparotomy. In recent years, the selective non-operative treatment has gained acceptance [44,45], mainly due to complication rates as high as 41% due to unnecessary laparotomies in patients with abdominal trauma [46,47]. Sonography, especially the protocol for FAST, and the CT-scan (only for stable patients) are generally accepted diagnostic tools for patients with abdominal gunshot injuries [45,48]. The diagnostic peritoneal lavage (DPL) can give a more or less accurate information about the presence of blood in the peritoneal cavity, but is used much less frequently in the evaluation of trauma patients because of the more sensitive and less invasive ultrasound examination. On the other hand sole ultrasound should not be the basis for decision making whether to operate or not [49].

There is general consensus that laparotomy is indicated in patients with abdominal gunshot injuries who are hemodynamically unstable or show signs of peritonitis or evisceration [44,48,50,51]. In hemodynamic stable patients with penetrating wounds on the left thoraco-abdominal region, laparoscopy is the preferred diagnostic tool with its specialty in detecting smaller diaphragmatic or intraabdominal injuries [52].

### Spinal injuries

**Figure 4:**
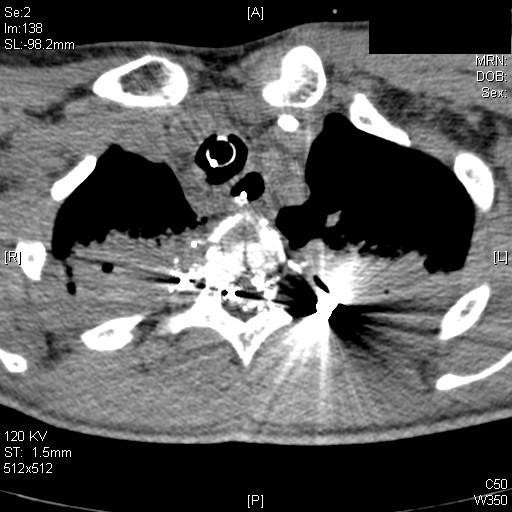
**CT-scan: Spinal gunshot with destruction of the spinal cord**. These injury caused a complete paraplegia and an unstable fracture of the vertebra.

Spinal gunshot injuries are usually not immediately life threatening. The primary diagnostic tool after clinical assessment of the neurological status should be a multislice helical CT scan, although CT scans may not be able to identify spinal injuries caused by indirect trauma as cavitation (Fig. 4). In these cases a magnetic resonance imaging (MRI) may offer further information; however due to the unknown load of ferromagnetical particles a MRI is associated with a residual risk. This risk depends on the material of the bullet: steel is more dangerous and responsible for more artefacts than lead [53]. Nonetheless, most authors conclude that the benefit of a MRI exceeds its risk in most cases [54].

The operative therapy of spinal gunshot injuries belongs in the second phase. Many patients could be treated conservatively. In general, unstable vertebral gunshot fractures are rare [55-57]. The classification and treatment strategies concerning vertebral stability are concordant to blunt fractures. The primary removal of projectiles is increasingly turned down. Concomitant neurological disorders cannot be improved in most cases [58] and the outcome of incomplete disorders is independent whether they are treated operatively or non operatively. But if the projectile is responsible for secondary neurological problems or infection it should be removed [59]. However, operative decompression of the spine seems only be potentially helpful in cases of incomplete or secondary progressive deficits if the damage is located between T12 and L4 [60]. Further, bullet fragments in the vertebral discs can induce lead poisoning and therefore should be extracted [61].

Considering the current literature admitting of steroids in cases of spinal gunshot injuries is not recommend [62-64]. There is consensus that an antibiotic prophylaxis should be admitted.

### Soft tissue injuries

Low-energy injuries are usually associated with minimal soft tissue damage and low risk of wound infection. Most of these injuries can be managed with superficial debridement and irrigation followed by a sterile dressing with or without antibiotics. Direct closure of the wound is not recommended; wounds are left to heal by secondary intention. Further, projectiles that cannot be palpated subcutaneously, should be left in situ since the risk of lead poisoning or infection is extremely low [65-67]^;^[68]. In contrast, high-energy and shotgun injuries are associated with severe soft tissue damage and require an aggressive debridement with several second-look surgeries. Excision of wound margins and wound track, as well as a careful removal of foreign material such as clothing and shotgun wadding are required [10]. Obviously healthy tissue should not be excised because several studies argue that an over-aggressive debridement leads to greater disability in the patient than that caused by the bullet [69,70].

### Bony injuries

In low-energy gunshot fractures, care is usually dictated by the bony injury because these fractures have similar personalities as closed fractures [4,6,71]. Unstable fractures require an appropriate method of surgical stabilization; those that can be controlled easily may be treated non-operatively [72].

High-energy gunshot fractures usually present with severe comminution with devitalized bone fragments and should be treated according to open fracture protocols [9,71]. The risk of infection and compartment syndrome in these injuries is extremely high and makes external fixation with or without fasciotomy the mainstay of primary fracture stabilization [73]. In civilians, most firearm injuries to the extremities are not life threatening and the need for amputation mostly depends on neurovascular, soft tissue, and bone injuries.

### Prophylactic measures

Although the belief that bullets are sterilized by the heat of firing is false [74,75], the administration of prophylactic antibiotics to patients with low-energy gunshot injuries has been debated [76-79]. Knapp et al. [80] showed that if an antibiotic treatment is desired, there is no difference in infection rates in low-energy gunshot fractures treated with intravenous antibiotics as compared with oral antibiotics. Dickey et al. [76] even reported no difference in the infection rates of patients with low-energy gunshot fractures treated with or without antibiotics, and Ordog et al. [81] showed that in a series of 3000 patients with low-energy gunshot wounds the overall infection rate of less than 2% was not reduced by the administration of antibiotic coverage. Risk factors for infection included delay in wound management, lack of adequate wound management, a wound size between 1-2 cm, and failure to comply with the instructions on wound care [81].

However, there is general consensus that gunshot injuries with bowel injury, penetrating craniocerebral injuries or high-energy gunshot injuries with moderate to severe soft tissue destruction require intravenous antibiotic treatment [6,79,82,83]. After penetrating gunshot injuries of the head broad spectrum antibiotics should be admitted as fast as possible.

Current guidelines recommend a single preoperative dose of prophylactic antibiotics with broad-spectrum aerobic and anaerobic coverage as a standard of care for trauma patients sustaining penetrating abdominal wounds. Absence of a hollow viscus injury requires no further administration [84]. The concepts for preventive antibiotic usage for penetrating chest trauma are controversial. Some authors showed benefits for antibiotic prophylaxis for patients from the insertion of a chest tube until its removal [85-87]. Other studies showed the same results for single shot therapy and prolonged antibiotics [88]. The recommendation for high-energy gunshot injuries with moderate soft tissue destruction is 48 hours intravenous administration of a first-generation cephalosporin. Penicillin must be added in patients with gross contamination and gentamicin may be added in the presence of severe soft tissue damage [79]. In special circumstances of grossly contaminated wounds, such as those with bowel communication or grossly dirty skin or clothing, we recommend the administration of a broad spectrum antibiotic for 1 to 2 weeks, although there continues to be no evidence that extending antibiotic prophylaxis beyond 24 hours is of benefit [89].

## Conclusion

The treatment strategy for patients with gunshot injuries to the torso mostly depends on the hemodynamic status of the patient. Whereas hemodynamically unstable patients require immediate operative measures like thoracotomy or laparotomy, hemodynamically stable patients might be treated with minor surgical procedures (e.g. chest tube) or even conservatively.

The treatment of craniocerebral gunshot injuries follows in general the guidelines for blunt injuries: Dura leakages should be closed and space consuming haematomas should be evacuated. Invasive measurement of the ICP can help to detect an increase at an early stage.

Spinal injuries often can be treated non-operatively. In cases of incomplete neurological deficits after injuries of the lower spine a decompression might be helpful. Admitting of steroids is generally not recommended.

Gunshot fractures should be treated following established fracture guidelines; closed fracture protocols should be used for low-energy gunshot injuries and open fracture protocols in high-energy gunshot wounds.

With the exception of craniocerebral, bowel, articular, or severe soft tissue injury, the use of antibiotics is controversial and may depend on the surgeon's preference.

## Conflict of interests

The authors declare that they have no competing interests.

## Authors' contributions

PL drafted the manuscript. MB and RO had great influence in the section of abdominal and thorax trauma. RW had added his experiences in antibiotic treatment. HCP has been head of an orthopedic surgery department in a large city in the US. He has wide experiences in treatment of gunshot injuries and gave practical advices for treatment. PK has already published several articles about treatment of gunshot fractures, and preclinical and emergency care of penetrating injuries. He was involved in all chapters and helped to draft the manuscript.

All authors read and approved the final manuscript.
